# Targeting HSPA1A in ARID2-deficient lung adenocarcinoma

**DOI:** 10.1093/nsr/nwab014

**Published:** 2021-01-30

**Authors:** Xue Wang, Yuetong Wang, Zhaoyuan Fang, Hua Wang, Jian Zhang, Longfu Zhang, Hsinyi Huang, Zhonglin Jiang, Yujuan Jin, Xiangkun Han, Shenda Hou, Bin Zhou, Feilong Meng, Luonan Chen, Kwok-Kin Wong, Jinfeng Liu, Zhiqi Zhang, Xin Zhang, Haiquan Chen, Yihua Sun, Liang Hu, Hongbin Ji

**Affiliations:** State Key Laboratory of Cell Biology, Shanghai Institute of Biochemistry and Cell Biology, Center for Excellence in Molecular Cell Science, Chinese Academy of Sciences, Shanghai 200031, China; State Key Laboratory of Cell Biology, Shanghai Institute of Biochemistry and Cell Biology, Center for Excellence in Molecular Cell Science, Chinese Academy of Sciences, Shanghai 200031, China; University of Chinese Academy of Sciences, Beijing 100049, China; State Key Laboratory of Cell Biology, Shanghai Institute of Biochemistry and Cell Biology, Center for Excellence in Molecular Cell Science, Chinese Academy of Sciences, Shanghai 200031, China; State Key Laboratory of Cell Biology, Shanghai Institute of Biochemistry and Cell Biology, Center for Excellence in Molecular Cell Science, Chinese Academy of Sciences, Shanghai 200031, China; State Key Laboratory of Cell Biology, Shanghai Institute of Biochemistry and Cell Biology, Center for Excellence in Molecular Cell Science, Chinese Academy of Sciences, Shanghai 200031, China; University of Chinese Academy of Sciences, Beijing 100049, China; Department of Pulmonary Medicine, ZhongShan Hospital, Fudan University, Shanghai 200032, China; State Key Laboratory of Cell Biology, Shanghai Institute of Biochemistry and Cell Biology, Center for Excellence in Molecular Cell Science, Chinese Academy of Sciences, Shanghai 200031, China; State Key Laboratory of Cell Biology, Shanghai Institute of Biochemistry and Cell Biology, Center for Excellence in Molecular Cell Science, Chinese Academy of Sciences, Shanghai 200031, China; University of Chinese Academy of Sciences, Beijing 100049, China; State Key Laboratory of Cell Biology, Shanghai Institute of Biochemistry and Cell Biology, Center for Excellence in Molecular Cell Science, Chinese Academy of Sciences, Shanghai 200031, China; State Key Laboratory of Cell Biology, Shanghai Institute of Biochemistry and Cell Biology, Center for Excellence in Molecular Cell Science, Chinese Academy of Sciences, Shanghai 200031, China; State Key Laboratory of Cell Biology, Shanghai Institute of Biochemistry and Cell Biology, Center for Excellence in Molecular Cell Science, Chinese Academy of Sciences, Shanghai 200031, China; State Key Laboratory of Cell Biology, Shanghai Institute of Biochemistry and Cell Biology, Center for Excellence in Molecular Cell Science, Chinese Academy of Sciences, Shanghai 200031, China; State Key Laboratory of Cell Biology, Shanghai Institute of Biochemistry and Cell Biology, Center for Excellence in Molecular Cell Science, Chinese Academy of Sciences, Shanghai 200031, China; State Key Laboratory of Cell Biology, Shanghai Institute of Biochemistry and Cell Biology, Center for Excellence in Molecular Cell Science, Chinese Academy of Sciences, Shanghai 200031, China; Division of Hematology and Medical Oncology, Laura and Isaac Perlmutter Cancer Center, New York University Langone Medical Center, New York, NY 10016, USA; College of Life Sciences, Qufu Normal University, Qufu 273165, China; Shanghai University of Medicine and Health Sciences, Shanghai Sixth People's Hospital East Campus, Shanghai 201306, China; Department of General Surgery, Shanghai Jiao Tong University Affiliated Sixth People's Hospital, Shanghai 200233, China; Department of Pulmonary Medicine, ZhongShan Hospital, Fudan University, Shanghai 200032, China; Department of Thoracic Surgery, Fudan University Shanghai Cancer Center, Shanghai 200032, China; Department of Thoracic Surgery, Fudan University Shanghai Cancer Center, Shanghai 200032, China; State Key Laboratory of Cell Biology, Shanghai Institute of Biochemistry and Cell Biology, Center for Excellence in Molecular Cell Science, Chinese Academy of Sciences, Shanghai 200031, China; State Key Laboratory of Cell Biology, Shanghai Institute of Biochemistry and Cell Biology, Center for Excellence in Molecular Cell Science, Chinese Academy of Sciences, Shanghai 200031, China; School of Life Science and Technology, Shanghai Tech University, Shanghai 200120, China

**Keywords:** lung adenocarcinoma, tumor suppressor, ARID2, HSPA1A

## Abstract

Somatic mutations of the chromatin remodeling gene ARID2 are observed in ∼7% of human lung adenocarcinomas (LUADs). However, the role of ARID2 in the pathogenesis of LUADs remains largely unknown. Here we find that ARID2 expression is decreased during the malignant progression of both human and mice LUADs. Using two *Kras^G12D^*-based genetically engineered murine models, we demonstrate that ARID2 knockout significantly promotes lung cancer malignant progression and shortens overall survival. Consistently, *ARID2* knockdown significantly promotes cell proliferation in human and mice lung cancer cells. Through integrative analyses of ChIP-Seq and RNA-Seq data, we find that *Hspa1a* is up-regulated by *Arid2* loss. Knockdown of *Hspa1a* specifically inhibits malignant progression of *Arid2*-deficient but not *Arid2*-wt lung cancers in both cell lines as well as animal models. Treatment with an HSPA1A inhibitor could significantly inhibit the malignant progression of lung cancer with ARID2 deficiency. Together, our findings establish ARID2 as an important tumor suppressor in LUADs with novel mechanistic insights, and further identify HSPA1A as a potential therapeutic target in ARID2-deficient LUADs.

## INTRODUCTION

Chromatin remodeling is known to play important roles in multiple physiological as well as pathological settings [1,2]. Conserved from yeast to human, the SWI/SNF (switch/sucrose nonfermenting) complex, as the essential component of chromatin remodelers, is involved in cell differentiation, proliferation and the DNA repair process [3,4]. The SWI/SNF complex, consisting of ∼15 subunits including lineage-specific variants, can slide the nucleosome along DNA in an adenosine triphosphate (ATP)-dependent manner and control lineage-specific gene expression via combinatorial assembling [2]. Loss of function of the SWI/SNF complex is potentially associated with disease malignant transformation [1]. Individual components of SWI/SNF complex are frequently mutated in cancer and the collective mutation rates for this complex vary among epithelial cancers, e.g. 75% in ovarian clear cell carcinoma [5], 57% in clear cell renal cell carcinoma [6], 40% in hepatocellular carcinoma [7], 34% in melanoma [8] and 35.12% in lung cancer [9].

BAF (Brg/Brahma-associated factors) and PBAF (Polybromo-associated BAF) are two variant forms of the SWI/SNF chromatin-remodeling complex. These two forms share many subunits but have also subtype specific subunits: BAF250 and hBRM are only found in BAF, whereas BAF180 and BAF200 are only found in PBAF [10]. BAF200, which is encoded by *ARID2*, is required for the function and selectivity of PBAF. Knockdown of ARID2 may affect the protein levels of other PBAF subunits as well as the function of PBAF in development and differentiation [11,12]. ARID2 is currently considered as a tumor suppressor gene since its nonsense mutations found in ∼10% melanoma samples are predicted to be loss-of-function mutations and lack the capability for DNA binding [8]. The inactivating mutation rate of ARID2 is ∼18.2% of HCV-associated hepatocellular carcinomas in the US and Europe [7,13]. In addition, ARID2 is also listed as one of the most frequently mutated genes after TP53, KRAS, EGFR, CDKN2A and STK11 (or LKB1) with an inactivating mutation rate ∼7.3% in lung adenocarcinomas (LUADs), the major subtype of lung cancer [14,15]. However, the contribution of ARID2 to the malignant progression of LUADs remains largely uncharacterized.

In this study, we found that ARID2 expression was decreased during the malignant progression of LUADs. Using autochthonous mouse models of lung cancer [16,17], we demonstrated a tumor suppressive role of ARID2 in LUADs. Moreover, we identified HSPA1A as a potential target for ARID2-deficient LUADs.

## RESULTS

### ARID2 expression is decreased during the malignant progression of LUADs

**Figure 1.**
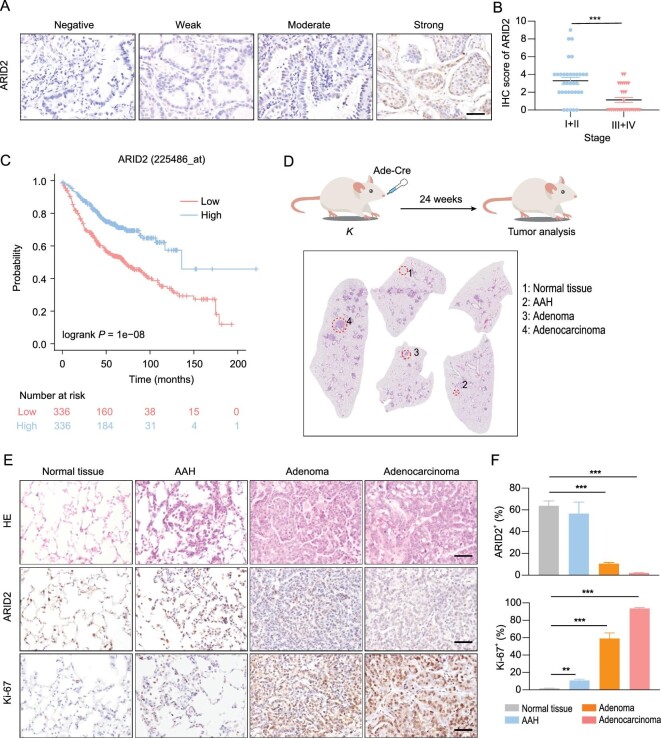
ARID2 expression is decreased during LUAD malignant progression. (A) ARID2 immunostaining in human LUAD. Representative photos for different expression levels. Scale bar: 50 μm. (B) Statistical analysis of ARID2 expression in 63 human LUADs with different stages. Stage I (n = 17), Stage II (n = 17), Stage III (n = 25) and Stage IV (n = 4). (C) Kaplan-Meier survival curve. (D) Schematic illustration of *Kras*-driven lung cancer mouse model and histopathological phenotype was shown. *K* mice at 6–8 weeks old were treated with 2 × 10^6^ p.f.u. Ade-Cre via nasal inhalation and analyzed at 24 weeks after Ade-Cre administration. (E–F) Representative HE and immunostaining (E) and quantification (F) of ARID2 and Ki-67 expression in *K* mice with different histopathological phenotypes. Scale bars: 50 μm. **P < 0.01, ***P < 0.001.

Through immunostaining analyses of 63 human LUAD samples (Fig. 1A), we found that ARID2 expression was significantly decreased with disease malignant progression (Fig. 1B). Moreover, we found that those patients with low ARID2 expression were associated with poor survival (Fig. 1C) (http://www.kmplot.com/lung) [18].

To evaluate the expression levels of ARID2 during the development and progression of LUADs *in vivo*, we employed a well-established autochthonous LUAD mouse model driven by *Kras^G12D^* mutant [16]. Lox-Stop-Lox *Kras^G12D/+^* (*K*) mice were treated with Ade-Cre through nasal inhalation to induce lung tumors as described previously [19]. After 24 weeks of Ade-Cre administration, the lungs of *K* mice exhibited multifocal and heterogeneous lesions, including atypical adenomatous hyperplasia (AAH, grade I), adenoma (grade II) and adenocarcinoma (grade III, Fig. 1D). We found that ARID2 expression was progressively decreased from AAH, adenoma, to adenocarcinoma in this model (Fig. 1E and F). Meanwhile, Ki-67 staining was gradually increased with disease malignant progression, consistent with an increased proliferation index from AAH to adenocarcinoma (Fig. 1E and F). These results indicate that ARID2 might act as a candidate tumor suppressor in LUADs.

### ARID2 deletion facilitates malignant progression of autochthonous lung cancer

Next, we determined the effects of ARID2 deletion *in vivo*. We found that *Arid2^fl/^^fl^* mice did not develop detectable lung tumors even after 70 weeks of Ade-Cre administration (Fig. S1A and B), indicating that ARID2 deletion alone is insufficient to drive lung tumor formation.

**Figure 2.**
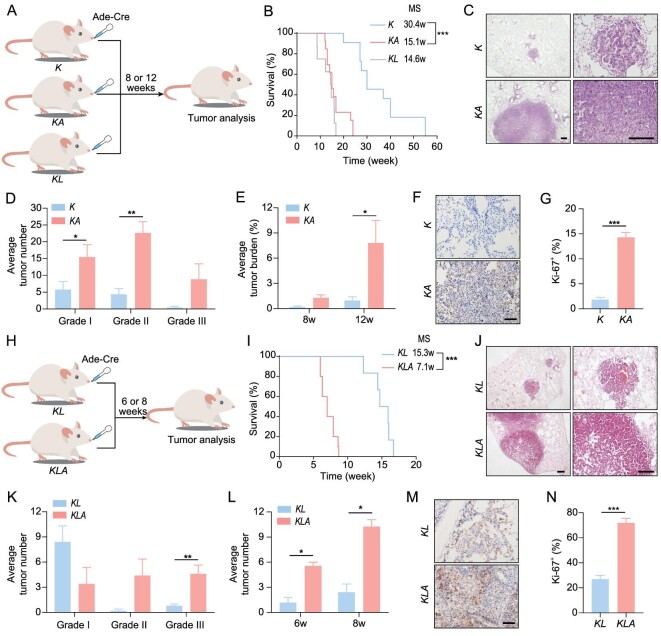
ARID2 depletion facilitates murine lung cancer malignant progression. (A) Schematic illustration of *K, KA* and *KL* mouse models. (B) Kaplan-Meier survival curves. MS: median survival time. (C) Representative histology of lung tumors from *K* and *KA* mice at 12 weeks post-Ade-cre treatment. Scale bar: 200 μm. (D and E) Quantification of tumor number (D) and burden (E). n = 5. (F) Representative Ki-67 immunostaining. Scale bar: 50 μm. (G) Quantification of Ki-67 immunostaining. (H) Schematic illustration of *KL* and *KLA* mouse models. (I) Kaplan-Meier survival curves. (J) Representative histology of lung tumors from *KL* and *KLA* mice at six weeks post-Ade-cre treatment. Scale bar: 200 μm. (K and L) Quantification of tumor number (K) and burden (L). n = 5. (M) Representative Ki-67 immunostaining. Scale bar: 50 μm. (N) Quantification of Ki-67 immunostaining. *P < 0.05, **P < 0.01, ***P < 0.001.

To evaluate the role of ARID2 in the pathogenesis of autochthonous mouse models of lung cancer, we first crossed the *Arid2^fl/^^fl^* mice to *K* mice to generate the *Kras^G12D/+^Arid2^fl/^^fl^* (*KA*) mice. We treated *K* or *KA* mice with Ade-Cre through nasal inhalation [19] and used the *Kras^G12D/+^Lkb1 ^fl^^/^^fl^* (*KL*) mice as a positive control (Fig. 2A), which is well-known for highly malignant tumors [17]. We confirmed *Lkb1* and *Arid2* deletion by polymerase chain reaction (PCR) genotyping (Fig. S1C). Notably, *KA* mice had much shorter survival than *K* mice, with a median time comparable to *KL* mice (Fig. 2B). Histopathological analyses confirmed that ARID2 deletion greatly promoted the malignant progression of lung cancer in *K* mice (Fig. 2C). Both tumor number and tumor burden were significantly increased in *KA* mice (Fig. 2D and E). Consistently, increased Ki-67 staining was observed in *KA* tumors (Fig. 2F and G).

We further crossed the *Arid2^fl/^^fl^* mice to *KL* mice and generated the *Kras^G12D/+^ Lkb1 ^fl^^/^^fl^ Arid2^fl/^^fl^* (*KLA*) cohort. The *KLA* or *KL* mice were then treated with Ade-Cre via nasal inhalation (Fig. 2H) and the efficiency of *Lkb1* and *Arid2* deletion was confirmed by PCR genotyping (Fig. S1C). The *KLA* mice had a much shorter survival than *KL* mice (Fig. 2I). After six weeks of Ade-Cre treatment, larger tumors were frequently observed in *KLA* mice compared to *KL* mice (Fig. 2J). Moreover, an increased number of grade II and III tumors and a decreased number of grade I tumors were observed in *KLA* mice (Fig. 2K). Consistently, increased tumor burden and Ki-67 staining were observed in *KLA* mice (Fig. 2L–N). All the *KLA* mice (5/5) developed metastases into lymph node, liver and chest wall as early as seven weeks post-Ade-Cre treatment (Fig. S2A). In contrast, there was no detectable metastasis in *KL* mice (0/5) at the same time point. Primary tumors and metastatic lesions kept proliferative and positive for the LUAD biomarker TTF1 (Fig. S2B). We further established a *KLA* tumor-derived primary cell line (hereafter refered to as *KLA* cells) (Fig. S2C) and found that ectopic expression of ARID2 significantly inhibited the migratory capability of *KLA* cells (Fig. S2D and E). These data strongly suggest a tumor-suppressive role for ARID2 in lung cancer malignant progression.

### Knockdown of ARID2 accelerates cell proliferation in human and mice lung cancer cells

**Figure 3.**
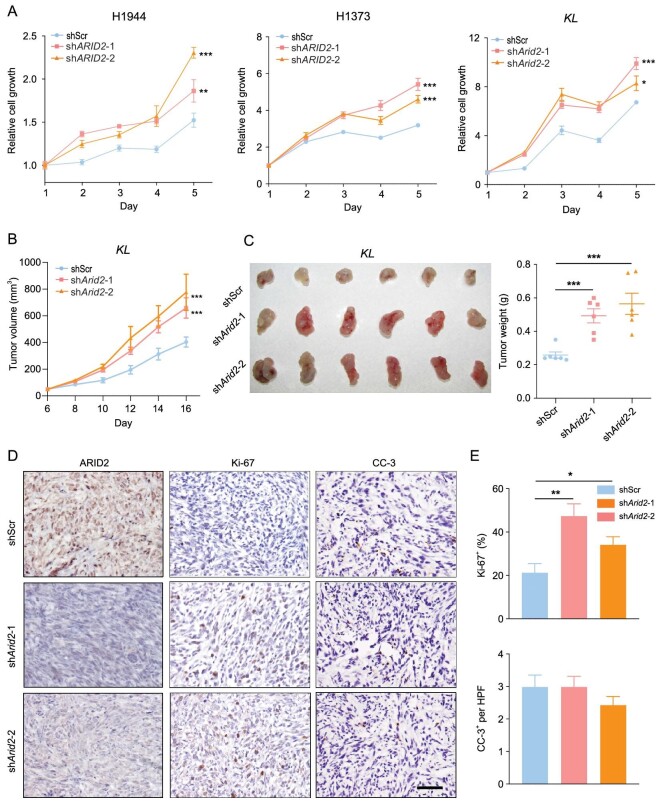
ARID2 knockdown accelerates human and mice lung cancer cell proliferation. (A) Relative growth curve of human LUAD cell lines H1944, H1373 and mouse *KL* cells with or without ARID2 knockdown. (B) Tumor growth of *KL* allografts with or without ARID2 knockdown. n = 6. (C) Photos and tumor weight of *KL* tumors with or without ARID2 knockdown. (D) Representative immunostaining of ARID2, Ki-67 and CC-3 in *KL* tumors with or without ARID2 knockdown. Scale bar: 50 μm. (E) Quantification of Ki-67 and CC-3 immunostaining. *P < 0.05, **P < 0.01, ***P < 0.001.

To evaluate the role of ARID2 *in vitro*, we depleted ARID2 in human LUAD cell lines H1944 and H1373 and found that ARID2 knockdown significantly promoted cell growth, which could be rescued by ARID2 re-expression (Fig. 3A and Fig. S3A and B). Next, we sought to confirm the function of ARID2 in primary cells derived from mouse tumors. Since it is difficult to establish primary cell lines from *K* tumors, we employed a *KL* tumor-derived primary cell line (hereafter refer to as *KL* cells) to perform subsequent *in vitro* and *in vivo* studies. Consistently, ARID2 knockdown accelerated *KL* cell proliferation, which could be reversed by ARID2 re-expression (Fig. 3A and Fig. S3A and B). Moreover, ARID2 knockdown significantly promoted *KL* tumor growth (Fig. 3B and C). Immunohistochemical analysis revealed that ARID2-knockdown tumors had increased Ki-67 positive staining compared with control counterparts (Fig. 3D and E). No substantial difference in the positive staining of cleaved-caspase-3 (CC-3) was observed between the ARID2-knockdown and control groups (Fig. 3D and E), indicating that ARID2 mainly suppresses proliferation of lung cancer cells. These data further solidify the role of ARID2 as a lung tumor suppressor.

### ARID2 knockout transcriptionally up-regulates *HSPA1A* expression potentially through a de-repression mechanism

ARID2 is a core component of the SWI/SNF chromatin-remodeling complex and regulates downstream gene transcription [11,20]. The ARID2-containing PBAF chromatin regulatory complex activates or represses gene transcription depending on different binding sites and/or co-factors [21,22]. To examine the possible mechanism by which ARID2 exerts its tumor suppressive function in LUADs, we performed RNA-Seq analysis to compare differentially expressed genes between *KA* versus *K* tumors and *KLA* versus *KL* tumors, and identified a list of genes consistently up-regulated in ARID2-deficient tumors (Table S1).

**Figure 4.**
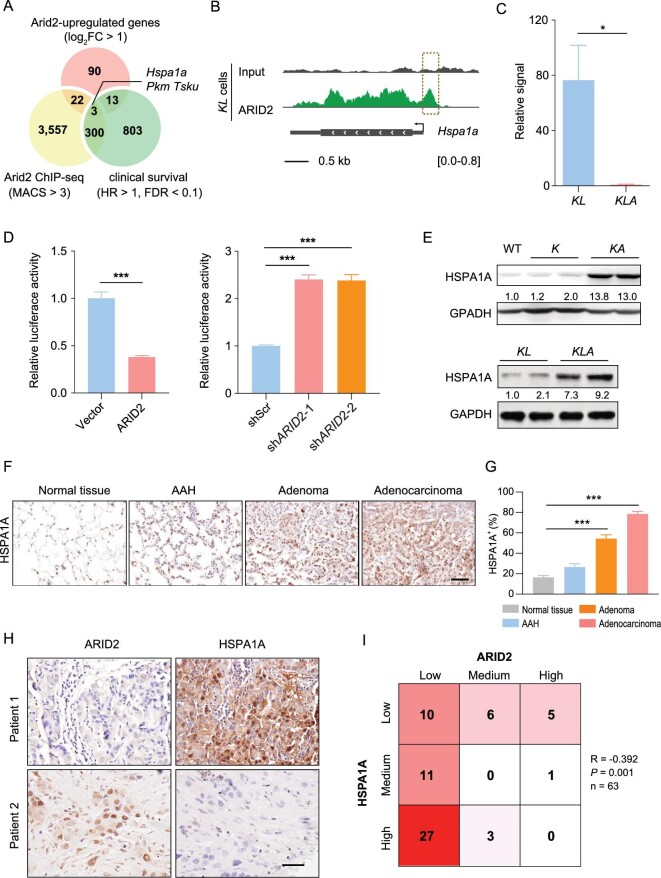
ARID2 loss transcriptionally up-regulates *HSPA1A* expression potentially through a de-repression mechanism. (A) Venn diagram of up-regulated genes in ARID2-loss tumors, ARID2 binding genes by ARID2 ChIP-seq and clinical survival-related genes from the TCGA-LUAD dataset. (B) ARID2 ChIP-seq of ARID2 binding signals along the promoter regions of *Hspa1a* genes in *KL* cells. Dashed box marks ARID2 binding peaks. (C) ChIP-qPCR assay confirmed the binding of ARID2 to the promoter region of *Hspa1a* gene in *KL* cells. Y axis shows the signal of *Hspa1a* promoter region relative to irrelevant primer. (D) Luciferase reporter assay in 293T cells with ARID2 overexpression or knockdown. (E) Western blot detection of HSPA1A expression in tumors from different mouse models. (F and G) Representative immunostaining (F) and quantification (G) of HSPA1A expression in *K* mice with different histopathological phenotypes at 24 weeks post-Ade-Cre treatment. Scale bars: 50 μm. (H) Representative immunostaining of ARID2 and HSPA1A in two human LUAD samples. Scale bar: 50 μm. (I) Correlation analysis of ARID2 and HSPA1A expression in human LUAD samples. *P < 0.05, ***P < 0.001.

To narrow down the list, we use *KL* cells for ARID2 ChIP-seq to identify genes directly regulated by ARID2 (Fig. 4A). Through integrative analyses of the consistently up-regulated genes, ARID2 ChIP-seq data and survival-related genes from the cancer genome atlas (TCGA)-LUAD dataset, we found three candidate genes including *Hspa1a, Pkm* and *Tsku* (Fig. 4A). *Hspa1a* belongs to the heat shock protein 70 (HSP70) family and is implicated in cancer development and drug sensitivity [23]. ARID2 ChIP-seq data revealed the distribution of ARID2 binding signals along the *Hspa1a* promoter region (Fig. 4B). Public human cell line ChIP-seq dataset also supported *HSPA1A* as a potential target of ARID2 (Fig. S4A). ChIP-qPCR in *KL* and *KLA* cells showed that ARID2 can bind to the promoter region of *Hspa1a* gene, which could be abolished upon ARID2 loss (Fig. 4C). We further performed luciferase reporter gene assay and found that ectopic ARID2 expression suppressed *Hspa1a* promoter activity, and vice versa (Fig. 4D and Fig. S4B). These data suggest that ARID2 loss promotes HSPA1A expression potentially through a transcriptional de-repression mechanism. Consistently, lung tumors with *Arid2* knockout (*KA* and *KLA*) showed higher levels of HSPA1A protein (Fig. 4E). Moreover, protein levels of HSPA1A progressively increased from AAH, adenoma, to adenocarcinoma in *K* mice after 24 weeks of Ade-Cre treatment (Fig. 4F and G). We further found that protein levels of ARID2 were inversely correlated with HSPA1A in 63 LUAD specimens (R = −0.392; *P* = 0.001) (Fig. 4H and I). We also observed a significant association between high HSPA1A expression and ARID2 mutation in 257 LUAD samples without HSPA1A genetic alterations from the TCGA database (Fig. S4C).

### HSPA1A knockdown preferentially suppresses the malignant progression of ARID2-deficient LUADs

**Figure 5.**
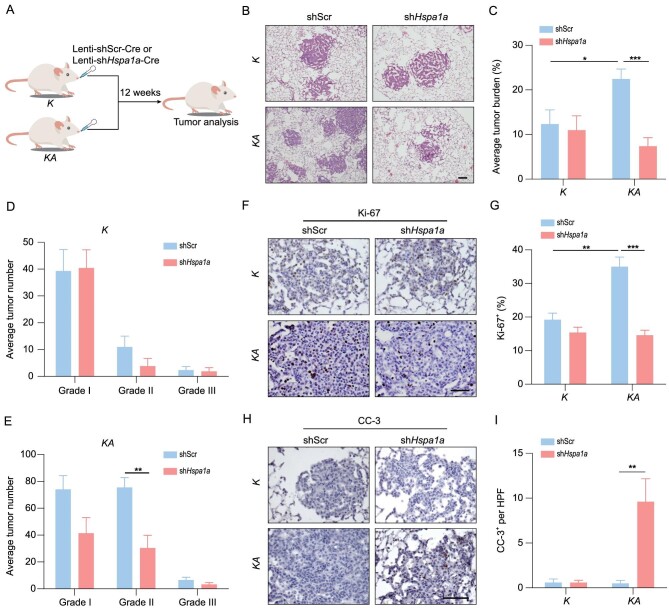
HSPA1A knockdown preferentially suppresses the malignant progression of ARID2-deficient LUADs. (A) Schematic illustration of HSPA1A knockdown in *K* and *KA* mouse models. (B) Representative histology of lung tumors from *K* and *KA* mice with or without HSPA1A knockdown at 12 weeks post-Lenti-cre treatment. Scale bar: 100 μm. (C–E) Quantification of tumor burden (C) and number (D and E) of *K* and *KA* mice with or without HSPA1A knockdown at 12 weeks post-Lenti-Cre treatment. n = 5. (F and G) Representative immunostaining (F) and quantification (G) of Ki-67 expression in tumors from *K* and *KA* mice with or without HSPA1A knockdown. Scale bar: 50 μm. (H and I) Representative immunostaining (H) and quantification (I) of CC-3 expression in tumors from *K* and *KA* mice with or without HSPA1A knockdown. Scale bar: 100 μm. *P < 0.05, **P < 0.01, ***P < 0.001.

To determine the role of HSPA1A depletion in the malignant progression of ARID2-deficient LUADs *in vivo*, we treated *K* or *KA* mice with lentivirus-mediated shRNA targeting Hspa1a through nasal inhalation (Fig. 5A). *Hspa1a* knockdown dramatically decreased tumor burden and number in *KA* mice without significant impact upon *K* mice (Fig. 5B–E and Fig. S5). Consistently, decreased Ki-67 staining and increased CC-3 staining were observed in HSPA1A-knockdown *KA* tumors (Fig. 5F–I). In contrast, no significant effect upon proliferation or apoptosis was observed in *K* tumors following HSPA1A knockdown (Fig. 5F–I). These data suggest that HSPA1A knockdown exerts superior antitumor effects in ARID2-deficient LUADs in mice.

### Genetic or pharmacological inhibition of HSPA1A specifically dampens ARID2-deficient lung tumor growth

**Figure 6.**
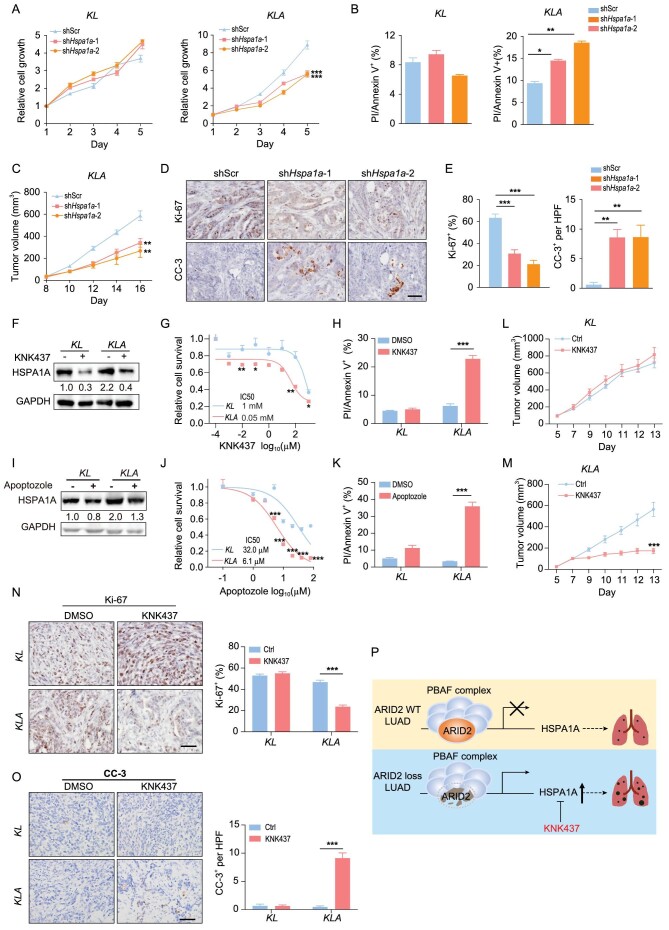
Genetic or pharmacological inhibition of HSPA1A specifically dampens ARID2-deficient lung tumor growth. (A) Relative growth curve of *KL* and *KLA* cells with or without HSPA1A knockdown. (B) Quantification of PI/Annexin V staining in *KL* and *KLA* cells with or without HSPA1A knockdown. (C) Tumor growth of *KLA* allografts with or without HSPA1A knockdown. n=5. (D and E) Representative immunostaining (D) and quantification (E) of Ki-67 and CC-3 expression in *KLA* tumors with or without HSPA1A knockdown. Scale bar: 50 μm. (F) Western blot detection of HSPA1A expression in *KL* and *KLA* cells with or without KNK437 treatment. (G) Dose response curves of KNK437 treatment in *KL* and *KLA* cells. (H) Quantification of the PI/Annexin V staining in *KL* and *KLA* cells with or without KNK437 treatment. (I) Western blot detection of HSPA1A expression in *KL* and *KLA* cells with or without Apoptozole treatment. (J) Dose response curves of Apoptozole treatment in *KL* and *KLA* cells. (K) Quantification of the PI/Annexin V staining in *KL* and *KLA* cells with or without Apoptozole treatment. (L and M) Tumor growth of *KL* (L) and *KLA* (M) allografts with or without KNK437 treatment. (N and O) Representative immunostaining and quantification of Ki-67 (N) and CC-3 (O) expression in *KL* and *KLA* tumors with or without KNK437 treatment. Scale bar: 50 μm. (P) Working model. ARID2 loss accelerates the malignant progression of LUAD potentially through HSPA1A. *P < 0.05, **P < 0.01, ***P < 0.001.

We further found that HSPA1A knockdown significantly inhibited the *KLA* cell proliferation, which could be reversed by HSPA1A re-expression (Fig. 6A and Fig. S6A and B). In contrast, knockdown of HSPA1A in *KL* cells did not apparently affect cell growth (Fig. 6A and Fig. S6A). We also found that ectopic HSPA1A expression promoted *KL* cell growth (Fig. S6C and D). Since HSPA1A has been implicated in cell apoptosis [24], we performed Annexin V/PI staining and found that HSPA1A knockdown specifically increased cell apoptosis in *KLA* cells but not in *KL* cells (Fig. 6B and Fig. S6E–G). Knockdown of HSPA1A also greatly suppressed the migratory capability of *KLA* cells (Fig. S6H and I). More importantly, HSPA1A knockdown significantly inhibited malignant progression of *KLA* allograft tumors (Fig. 6C and Fig. S6J and K). Decreased Ki-67 staining and increased CC-3 staining were observed in HSPA1A-knockdown groups (Fig. 6D and E). These findings suggest that ARID2-deficient cancer cells preferentially rely on HSPA1A for cell proliferation, survival and migration, which provides the vulnerability for therapeutic targeting.

KNK437 was reported as a specific HSPA1A inhibitor which is well tolerated in immuno-compromised mice [25]. We found that KNK437 treatment decreased HSPA1A protein levels *in vitro* and *in vivo* (Fig. 6F and Fig. S9A). Moreover, the IC50 value of KNK437 was significantly lower in *KLA* cells (0.05 mM) than *KL* cells (1 mM) (Fig. 6G). Consistent with the effects of HSPA1A knockdown, KNK437 treatment triggered overt cell apoptosis in *KLA* cells (Fig. 6H and Fig. S9B). These observations were further confirmed using another HSPA1A-specific inhibitor apoptozole [26] (Fig. 6I–K, and Fig. S9C).

Through analysis of the LUAD-TCGA database, we noticed that ARID2 mutations were almost evenly distributed along its open reading frame (ORF) region (Fig. S7A), indicative of the lack of hotspot mutations. COSMIC data showed that the human LUAD cell line H23 harbored an ARID2 mutation (452M>V). The IC50 of KNK437 in H23 cells was ∼0.14 mM (Fig. S7B), which was much lower than *KL* cells and comparable to *KLA* cells (Fig. 6G). The ARID2 M452V mutation was a loss-of-function mutation, as ectopic expression of wild-type ARID2, but not ARID2^M452V^ mutant, suppressed the HSPA1A expression and inhibited cell proliferation (Fig. S8A–C). Furthermore, either KNK437 treatment or HSPA1A knockdown preferentially suppressed the growth of *KLA* cells expressing ARID2^M452V^ mutant (Fig. S8D and E). Additionally, human liver cancer cell line SNU-398 (ARID2 mutant) was more sensitive to KNK437 than HepG2 (ARID2 wild-type) (Fig. S7B). These observations indicate that cancer cell lines harboring ARID2 mutations might be vulnerable to HSPA1A inhibition.

Importantly, KNK437 treatment greatly inhibited the growth of *KLA* allograft tumors without significant impact upon *KL* counterparts (Fig. 6L and M and Fig. S9D). Consistently, decreased Ki-67 staining and increased CC-3 staining were observed in KNK437-treated *KLA* tumors (Fig. 6N and O). These findings suggest that pharmacological inhibition of HSPA1A selectively suppresses ARID2-deficient lung tumor growth.

## DISCUSSION

Here we demonstrate that ARID2 acts as an important tumor suppressor gene in LUADs and reveal that ARID2 depletion induces HSPA1A expression potentially through a transcriptional de-repression mechanism. Importantly, genetic and pharmacological inhibition of HSPA1A exhibit impressive therapeutic effects in ARID2-deficient tumors, suggesting that targeting HSPA1A may be an effective strategy in ARID2-mutant LUADs (Fig. 6P).

High frequency of ARID2 mutation has been documented in different types of human cancers including lung cancer [15,27,28]. Loss-of-function mutations of ARID2 have been reported to be observed in ∼7.3% of LUADs [15]. However, the role of ARID2 in LUAD development remains largely unknown. Using clinical specimens and autochthonous mouse models of lung cancer, we provide strong evidence to illustrate a tumor suppressive role of ARID2 in LUADs.

In an attempt to search for the molecular events involved in mediating the effect of ARID2 depletion, we find that HSPA1A is one of the significantly up-regulated genes in ARID2-deficient lung tumors. Through RNA-seq, ChIP-seq, ChIP-qPCR and luciferase reporter assay, we demonstrate that ARID2 depletion transcriptionally up-regulates *Hspa1a* expression potentially through a transcriptional de-repression mechanism. The inverse correlation between ARID2 and HSPA1A expression is also observed in human LUADs. Importantly, genetic or pharmacological inhibition of HSPA1A preferentially inhibits malignant progression of ARID2-deficient LUADs in autochthonous genetically engineered murine models and allograft models. These findings not only support an essential role for HSPA1A in mediating the effects of ARID2 loss, but also identify HSPA1A as a potential vulnerable target in ARID2-deficient LUADs.

HSP70 proteins play essential roles in regulation of correct protein folding and maintenance of protein homeostasis [29]. These proteins enhance cell survival following a multitude of stresses, including elevated temperature, hypoxia, oxidative stress, altered pH, heavy metals and others [30]. The pro-survival role of HSP70 proteins is related to their ability to buffer the toxicity of denatured and misfolded proteins that accumulate during stress [24]. The HSP70 family consists of at least eight members with molecular chaperones of ∼70 kDa in size [29]. High expression of HSP70 has been correlated with poor prognosis in cancers of the liver [31], prostate [32], colon [33] and lung [34]. Multiple inhibitors have been designed to target the enzymatic activity of HSP70 and/or its interaction with important co-chaperones [35], and some of them have been evaluated as anticancer agents in pre-clinical or clinical trials [36–38]. Although we cannot rule out the potential involvement of other genes in ARID2 loss-mediated LUAD progression, our findings of the superior efficacy of HSP70 inhibitors in ARID2-deficient tumors clearly support the functional importance of HSPA1A in this setting and provide a potential new therapeutic strategy for clinical management of lung cancer with this subtype.

## METHODS

### Mice cohorts and treatment

Mice were housed in a specific pathogen-free environment at the Shanghai Institute of Biochemistry and Cell Biology and treated in accordance with protocols conforming to the ARRIVE guidelines and approved by the Institutional Animal Care and Use Committee of the Shanghai Institutes for Biological Sciences, Chinese Academy of Sciences (approval number: SIBCB-S215-2101-008]. *Kras^G12D/+^* and *Lkb1^fl^^/^^fl^* mice were originally generously provided by Dr T. Jacks and Dr R. Depinho, respectively. The *Arid2^fl/^^fl^* transgenic mice were previously described [39]. *K, KA, KL* and *KLA* mice at 6–8 weeks old were treated with 2 × 10^6^ p.f.u. Ade-Cre via nasal inhalation and analyzed at different time points.

### Statistical analysis

The significance of differences was determined using the one-way ANOVA or Student's t-test (two-sided). *P* < 0.05 is considered to be statistically significant. The Kendall's tau correlation analysis was used to analyze the correlation between ARID2 and HSPA1A expression in human LUAD samples. Data were represented as mean ± standard error of the mean unless otherwise indicated. All statistical analyses were carried out using GraphPad Prism 5 software. For more methods, see Supplementary Data.

## Supplementary Material

nwab014_Supplemental_File
