# Supplementary material for: Targeting HSPA1A in ARID2-deficient lung adenocarcinoma
Source: Natl Sci Rev. 2021 Jan 30;8(10):nwab014. doi: 10.1093/nsr/nwab014 (PMC8566174; doi:10.1093/nsr/nwab014)
Supplement: nwab014_Supplemental_File [file nwab014_supplemental_file.pdf]

SUPPLEMENTARY DATA

Targeting HSPA1A in ARID2-deficient lung adenocarcinoma

Xue Wang[1,†], Yuetong Wang[1,2,†], Zhaoyuan Fang[1,†], Hua Wang[1], Jian Zhang[1,2], Longfu Zhang[3], Hsinyi Huang[1], Zhonglin Jiang[1,2], Yujuan Jin[1], Xiangkun Han[1], Shenda Hou[1], Bin Zhou[1], Feilong Meng[1], Luonan Chen[1], Kwok-Kin Wong[4], Jinfeng Liu[5], Zhiqi Zhang[6,7], Xin Zhang[3], Haiquan Chen[8], Yihua Sun[8,*], Liang Hu[1,*], Hongbin Ji[1,9,*]

SUPPLEMENTARY METHODS

Cell culture

H1944, H1373 and H23 cell lines were purchased from ATCC and free of mycoplasma contamination. HepG2 cell line was a gift from Dr. Lijian Hui, Chinese Academy of Sciences (Shanghai, China). SNU-398 cell line was a gift from Dr. Daming Gao, Chinese Academy of Sciences (Shanghai, China). Cells were grown in DMEM or RPMI-1640 (HyClone, Logan, UT, USA) with 8% FBS (Gibco, Rockville, MD, USA). Primary cell lines derived from *KL* or *KLA* tumors (hereafter refer to as *KL* or *KLA* cells, respectively) were grown in DMEM (HyClone) with 8% FBS (Gibco) free of mycoplasma contamination and passaged every other day as previously reported (1).

Cell proliferation assay

Cells were seeded in sextuplicate in 96-well plates and stained with 3-(4,5-dimethyl-2-thiazolyl)-2,5-diphenyl-2-H-tetrazolium bromide (MTT) and assessed with Epoch multi-volume spectrophotometer system (570nm/630nm) at indicated time points.

Cell survival assay

Cells were seeded in quadruplicate in 96-well plate overnight, treated with indicated doses of various compounds 24 hrs later and cultured for another 72 hrs. Cells were then stained with MTT and assessed with Epoch multi-volume spectrophotometer system (570nm/630nm).

In vivo allograft studies

For *in vivo* allograft assay, specific-pathogen-free male BALB/c nude mice were randomized blindly into control and experimental groups. 5×10^5 cells were subcutaneously injected into the flank of nude mice until palpable tumors formed. Tumors were monitored every other day and tumor volume was calculated based on the volume of ellipsoid: tumor volume (mm^3) = (longer measurement × shorter measurement^2)/2. For the KNK437 treatment assay, mice were randomized blindly

into two groups treated with 200 mg/kg KNK437 or with equal volume of oil daily via intra-peritoneal injection.

Gene knockdown and overexpression

For gene knockdown study, lentivirus-mediated delivery of shRNAs targeting ARID2 and HSPA1A were performed as described previously (2). The shRNAs were packaged in lentiviral particles by co-transfection with packaging plasmids into HEK-293T cells and the filtered cell culture supernatant was then used to infect cells. The shRNA sequences are listed in the Table S2.

For gene overexpression study, the pCDH vector containing the target gene and packaging plasmids were introduced into HEK-293T cells for the production of lentivirus. The filtered cell culture supernatant was then used to infect target cells. The transfected cells were expanded for follow-up experiments.

Western blot assay

Cells or tissue lysates were prepared and subjected to western blot analysis with the following primary antibodies: HSPA1A (10995-1-AP, ProteinTech, Chicago, IL, USA), human ARID2 (GTX129444, GeneTex, Irvine, CA, USA), mouse ARID2 (SC98299X, Santa Cruz Biotechnology, Santa Cruz, CA, USA), GAPDH (AC002, ABclonal, Hubei, China), β-actin (AC026, ABclonal, Hubei, China).

Cell apoptosis assay

Cancer cells were stained using the PI/Annexin V double staining kit (C1062, Beyotime, Haimen, China) or PI/RNase staining buffer (550825, BD Biosciences, San Jose, CA, USA) and FACS was performed using the BD LSRII flow cytometer. FACS data was analyzed using FlowJo (TreeStar, Ashland, OR, USA).

Real-time PCR

Total RNAs were prepared and retrotranscribed into first-strand cDNA using the first-strand synthesis system following the manufacturer's protocol (Invitrogen, Carlsbad, CA). Real-time PCR was performed using ABI 7500 sequence detection system (Perkin Elmer Life Sciences, Shelton, CT, USA). Primer sequences are listed in Table S2.

Immunohistochemical staining

Immunostaining was performed as previously described (3). Immunostaining analysis was independently performed by two expert pathologists who were blinded to the experimental groups. Expression of ARID2 was using the German immunoreactive score (IRS) as described previously (4). Briefly, the staining intensity was graded as "0" (no staining), "1" (weakly stained), "2" (moderately stained), or "3" (strongly stained) and the staining extent was graded as "0" (<5%), "1" (5%–25%), "2" (25%–50%), "3" (50%–75%,), or "4" (>75%). The scores of the staining intensity and the staining extent were multiplied to give a final staining score of 0~12. Antibodies against the HSPA1A (10995-1-AP, ProteinTech), Ki-67 (NCL-Ki67p, Leica Biosystems, Wetzlar, Germany), Cleaved Caspase-3 (CST-9664, Cell Signaling Technologies, Danvers, MA, USA), human ARID2 (GTX129444, GeneTex) and mouse ARID2 (SC 98299X, Santa Cruz) were used.

Transwell migration assay

For cell migration, 1×10^5 cells were plated onto Transwell filters with 8-mm pores, a 24-well plate chamber insert (Corning, NY, USA) The top of the insert was supplemented with serum-free medium, while the bottom was supplemented with DMEM with 8% FBS. Cells were incubated for 12 hours and fixed with 4% paraformaldehyde for 15 minutes. After washing with PBS, cells at the top of the insert were scraped with a cotton swab. Cells adherent to the bottom were stained with hematoxylin for 1 minute and then washed 3 times with double-distilled H_2O. The positively stained cells on the underside of the filters were photographed and examined under the microscope.

ChIP-seq and ChIP-qPCR

Cells were cross-linked with 1% formaldehyde for 5 min at room temperature, lysed by SDS Lysis buffer and sonicated to generate DNA fragments with an average size of 500 bp. After pre-clearing with Protein G beads, antibodies against ARID2 (SC 98299X, Santa Cruz), RNA polymerase II (SC 900, Santa Cruz) or control IgG was added to cell lysate and incubated at 4℃overnight. DNA crosslinked with ARID2 was pulled down with Protein G beads, washed and purified with minElute PCR purification kit (NO. 28004, QIANGEN, Duesseldorf, Germany). Aliquots of

ChIP-enriched DNA and whole-cell lysate DNA were subjected to sequencing and qPCR analyses.

RNA sequencing and data analysis

Arid2 loss and control RNA samples were obtained from both *K* and *KL* mice tumors. The numbers of replicates were one and three for *K* and *KL* backgrounds, respectively. The library preparation and sequencing were performed according to the standard Illumina RNA-seq protocol (NovaSeq 6000, Berry Genomics, Inc.). Raw sequencing reads were first quality filtered with the following criteria: N% > 3%, or fraction of low quality bases (< 20) above 50%, or matching of known adapter sequences (>= 8bp) in any reads. RNA-seq reads were mapped to the current mouse genome (mm10) with the STAR aligner (v2.6.0c) and counted with featureCounts (v1.6.4) for each gene. Reads per million (RPM) values after log2 transformation were used for downstream analysis. For *K* background data with a single replicate, log2 fold changes was used to determine differential expression. For *KL* background data with three replicates, differential expression was evaluated with the Student's *t* test (two-sided). Multiple testing *P* values were then corrected with the widely used 'q value' method (v2.10.0) (5).

ChIP-seq data analysis

ChIP-seq reads were aligned to the mm10 genome with bwa (v0.7.15). The uniquely and properly mapped read pairs were retained after removal of duplicated reads. Binding peaks was called with MACS2 (v2.1.1) against the input sample (default parameters) and annotated with CEAS (v1.0.2). Public ChIP-seq datasets for ARID2 (SRX3468024, SRX3468012) were downloaded from NCBI SRA, mapped to hg19, and analyzed for binding peaks similarly (6, 7).

Human LUAD dataset analysis

Survival analysis of the full spectrum of patients with LUAD was performed based on ARID2 expression status using the online Kaplan Meier-plotter analysis tool (8). We performed survival analysis using the Affymetrix ID 225486_at for *ARID2* gene symbol. The median value was chosen as the cut-off for high and low ARID2

expression. The TCGA-LUAD dataset was employed to screen genes with an increased risk on patient survival (HR>1 and FDR<0.1).

Tumor samples with ARID2 mutations including both point mutations and fragment deletions were compared with other ARID2 wild-type samples for differential expression gene analysis. Since the expression of downstream genes could be affected by other mechanisms such as copy number variations (9), we restricted our analysis on samples with a neutral copy number of these downstream genes. For HSPA1A, 257 lung adenocarcinoma samples are with a neutral copy number, among which 221 are ARID2 wild-type and 36 are ARID2 mutant. To evaluate differential expression, Wilcoxon rank sum test was used with a *P* value threshold at 0.05.

Human lung cancer specimen analysis

The study cohort containing 63 LUAD tumor samples from Shanghai Cancer Center of Fudan University was collected and used for immunostaining of ARID2 and HSPA1A for correlation analysis. Patients were excluded from the study cohort with the following exclusion criteria: previously received any anticancer therapy; impaired heart, lung, liver or kidney function; previous malignant disease. All tumor specimens were taken at the time of surgical resection. Written informed consent was obtained from all patients and this study was conducted in accordance with the ethical standards set out in the WMA Declaration of Helsinki and the Department of Health and Human Services Belmont Report and approved by the institutional review committee of Shanghai Cancer Center of Fudan University.

REFERENCES

1. Li, F, Han, X, Li, F*, et al.* LKB1 Inactivation Elicits a Redox Imbalance to Modulate Non-small Cell Lung Cancer Plasticity and Therapeutic Response. *Cancer Cell*2015. 698-711.

2. Gao, Y, Zhang, W, Han, X*, et al.* YAP inhibits squamous transdifferentiation of Lkb1-deficient lung adenocarcinoma through ZEB2-dependent DNp63 repression. *Nat Commun*. 2014; **5**: 4629.

3. Han, X, Li, F, Fang, Z*, et al.* Transdifferentiation of lung adenocarcinoma in mice with Lkb1 deficiency to squamous cell carcinoma. *Nat Commun*. 2014; **5**: 3261.

4. Tang, L, Tan, YX, Jiang, BG*, et al.* The prognostic significance and therapeutic potential of hedgehog signaling in intrahepatic cholangiocellular carcinoma. *Clin Cancer Res*. 2013; **19**(8): 2014-24.

5. Storey, JD, Tibshirani, R. Statistical significance for genomewide studies. *Proc Natl Acad Sci U S A*. 2003; **100**(16): 9440-5.

6. McBride, MJ, Pulice, JL, Beird, HC*, et al.* The SS18-SSX Fusion Oncoprotein Hijacks BAF Complex Targeting and Function to Drive Synovial Sarcoma. *Cancer Cell*. 2018; **33**(6): 1128-41.e7.

7. Mahat, DB, Salamanca, HH, Duarte, FM*, et al.* Mammalian Heat Shock Response and Mechanisms Underlying Its Genome-wide Transcriptional Regulation. *Molecular cell*. 2016; **62**(1): 63-78.

8. Gyorffy, B, Surowiak, P, Budczies, J*, et al.* Online survival analysis software to assess the prognostic value of biomarkers using transcriptomic data in non-small-cell lung cancer. *PloS one*. 2013; **8**(12): e82241.

9. Stranger, BE, Forrest, MS, Dunning, M*, et al.* Relative impact of nucleotide and copy number variation on gene expression phenotypes. *Science (New York, NY)*. 2007; **315**(5813): 848-53.

SUPPLEMENTARY FIGURE LEGENDS

Fig. S1. ARID2 deletion alone is insufficient to induce detectable lung tumor.

(A) A scheme showing the strategy of *Arid2* $^{fl/fl}$ mouse model experimental design.

(B) Representative histology of lungs from *WT* and *Arid2* $^{-/-}$ mice at 70 weeks post Ade-Cre treatment. Scale bar: 500 μm.

(C) Genotyping PCR confirmed the efficiency of *Lkb1* and *Arid2* deletion in *KA, KL and KLA* GEMMs.

Fig. S2. ARID2 depletion promotes the metastasis of lung cancer in the *KL* mouse model.

(A) Representative histology of metastatic tumors from *KLA* mice at 6 weeks post Ade-cre treatment. Upper scale bar: 200 μm, lower scale bar: 80 μm.

(B) Immunostaining of Ki-67 and TTF1 in both primary lung tumors and metastatic lesions from *KLA* mice. Scale bar: 200 μm.

(C) Genotyping PCR confirmed the efficiency of *Arid2* knockout in *KLA* cells.

(D) Representative images of migrated *KLA* cells with or without ARID2 overexpression in a Transwell assay. Scale bars: 200 μm.

(E) Quantification of cell migration.

***P < 0.001.

Fig. S3. ARID2 knockdown promotes lung cancer cell growth.

(A) Western blot detection of ARID2 in indicated human lung cancer cell lines and mouse *KL* lung cancer cell lines with or without ARID2 knockdown or with ARID2 re-expression. β-actin serves as the internal control.

(B) Relative growth curve of indicated cell lines with or without ARID2 knockdown or with ARID2 re-expression.

*$P < 0.05$, **$P < 0.01$, ***$P < 0.001$.

Fig. S4. ARID2 binds to *HSPA1A* promoter and downregulates its expression.

(A) The distribution of ARID2 and input peaks along the promoter region of *HSPA1A* gene in ASKA cells was shown.

(B) Western blot detection of ARID2 overexpression or knockdown efficiency in 293T cells. GAPDH serves as an internal control.

(C) The correlation between HSPA1A mRNA level and ARID2 mutations in 257 LUAD samples without HSPA1A genetic alterations from the TCGA database.

*$P < 0.05$

Fig. S5. Immunohistochemical validation of HSPA1A knockdown.

Representative immunostaining of HSPA1A in *K* or *KA* mouse models with or without HSPA1A knockdown. Scale bar: 50 μm.

Fig. S6. HSPA1A knockdown specifically dampens ARID2-deficient lung tumor growth.

(A) Western blot detection of HSPA1A levels in *KL* and *KLA* cells with or without HSPA1A knockdown or with HSPA1A re-expression. GAPDH served as the internal control.

(B) Relative growth curve of *KLA* cells with or without HSPA1A knockdown or with HSPA1A re-expression.

(C) Western blot detection of HSPA1A levels in *KL* cells with or without HSPA1A overexpression. GAPDH served as the internal control.

(D) Relative growth curve of *KL* cells with or without HSPA1A overexpression.

(E) Representative images of PI/Annexin V staining in *KL* and *KLA* cell*s* with or without HSPA1A knockdown.

(F) Representative images of PI/Annexin V staining in *KLA* cells with or without HSPA1A knockdown or with HSPA1A re-expression.

(G) Quantification of cell apoptosis.

(H) Representative images of migrated *KLA* cells with or without HSPA1A knockdown or with HSPA1A re-expression in a Transwell assay. Scale bars: 200 μm.

(**I)** Quantification of cell migration.

(J) Tumor photos of the *KLA* cells with or without HSPA1A knockdown. n=6.

(K) Immunostaining of HSPA1A in *KLA* tumors with or without HSPA1A knockdown. Scale bar: 50 μm.

**P* < 0.05, ***P* < 0.01, ****P* < 0.001.

Fig. S7. Targeting HSPA1A is a potential therapeutic strategy in cancer cells with actual ARID2 mutation.

(A) ARID2 mutation profiles in LUAD and LUAD-derived cell lines.

(B) Dose response curves of KNK437 treatment in HepG2, SNU-398 and H23 cells.

Fig. S8. ARID2 M452V mutation is a loss-of-function mutation.

(A) Real-time PCR detection of ARID2 levels in *KLA* cells with wild-type ARID2 or $ARID2^{M452V}$ mutant overexpression.

(B) Western blot detection of HSPA1A levels in *KLA* cells with wild-type ARID2 or $ARID2^{M452V}$ mutant overexpression. GAPDH served as the internal control.

(C) Relative growth curve of *KLA* cells with wild-type ARID2 or $ARID2^{M452V}$ mutant overexpression.

(D) Relative growth of *KLA* cells with wild-type ARID2 or $ARID2^{M452V}$ mutant overexpression following KNK437 treatment.

(E) Relative growth of *KLA* cells with wild-type ARID2 or $ARID2^{M452V}$ mutant overexpression following HSPA1A knockdown.

$^{*}P < 0.05$, $^{**}P < 0.01$, $^{***}P < 0.001$.

Fig. S9. Pharmacological inhibition of HSPA1A specifically dampens ARID2-deficient lung tumor growth.

(A) Immunostaining of HSPA1A in *KL* or *KLA* tumors with or without KNK437 treatments. Scale bar: 50 μm.

(B) Representative results of Annexin V/ PI staining in *KL* and *KLA* cells with or without KNK437 treatment.

(C) Representative results of Annexin V/ PI staining in *KL* and *KLA* cells with or without Apoptozole treatment.

(D) Tumor photos of the *KL* and *KLA* tumors with or without KNK437 treatments. n=6.

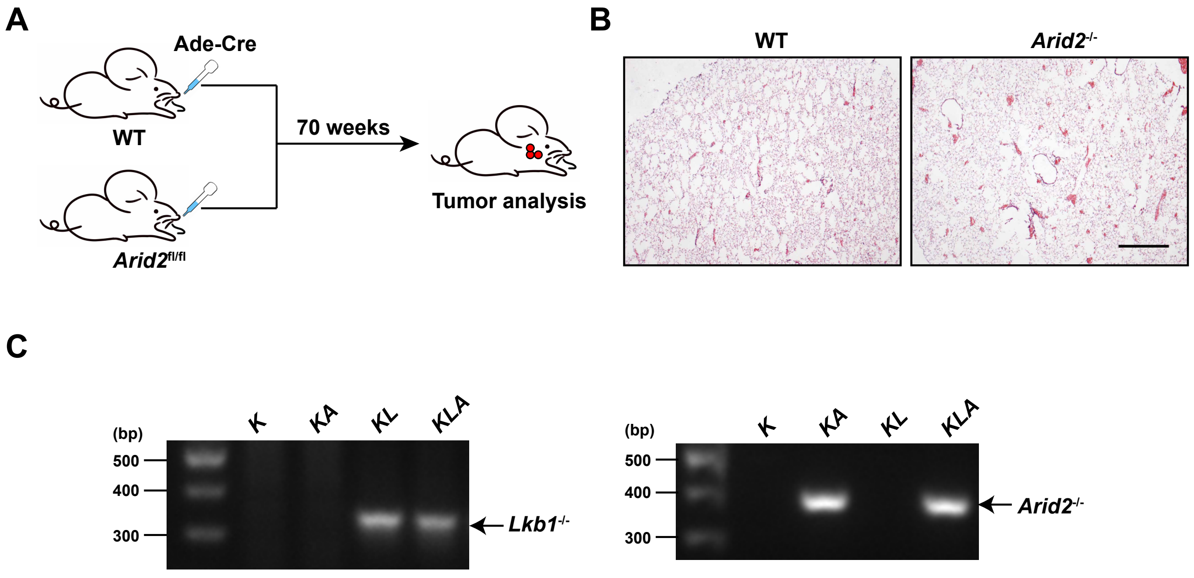
A
Ade-Cre
WT
Arid2fl/fl
70 weeks
Tumor analysis
B
WT
Arid2-/-
C
(bp)
500
400
300
K
KA
KL
KLA
Lkb1-/-
(bp)
500
400
300
K
KA
KL
KLA
Arid2-/-

Figure S1

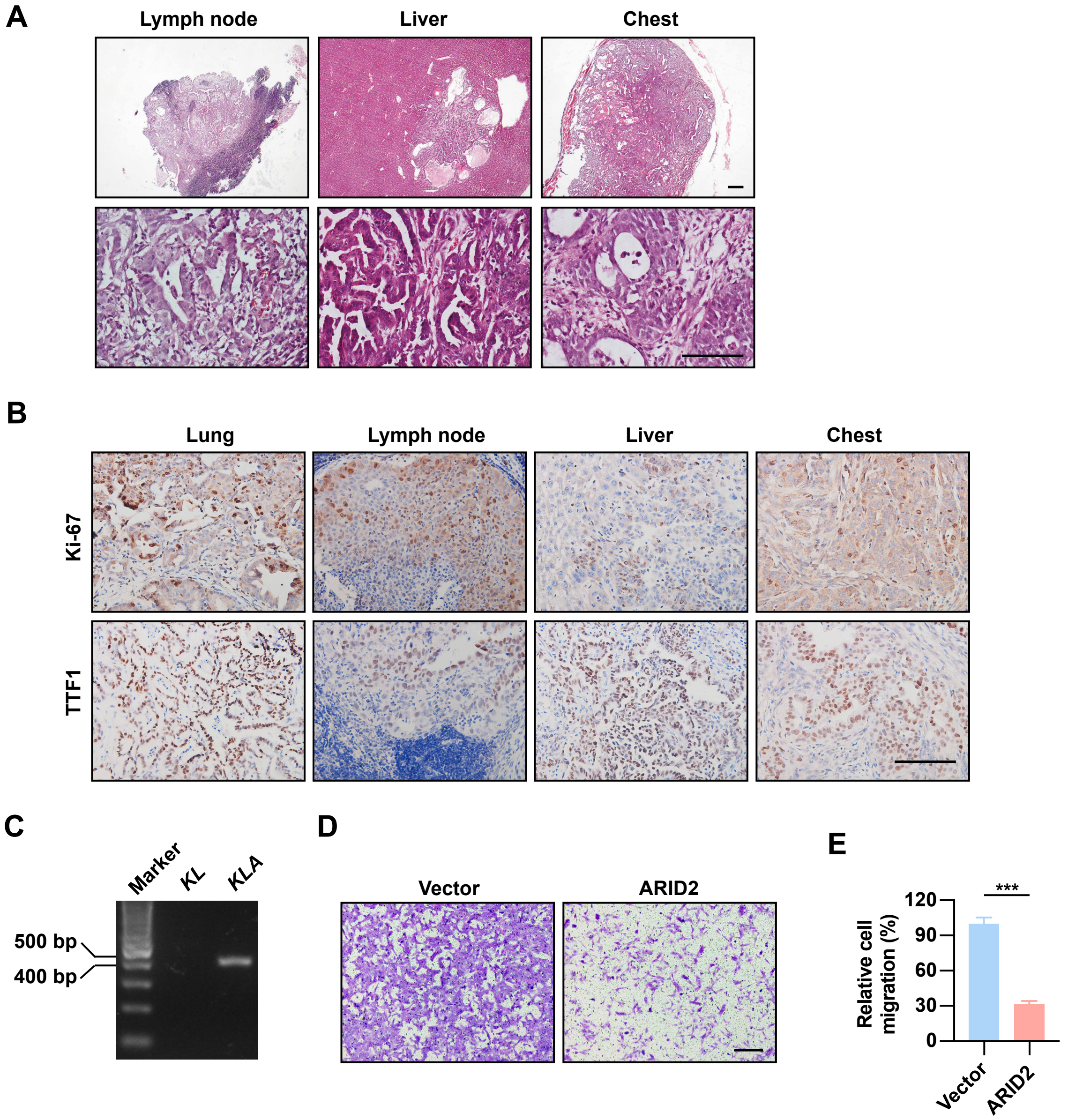
A
Lymph node
Liver
Chest
B
Lung
Lymph node
Liver
Chest
Ki-67
TTF1
C
Marker
KL
KLA
500 bp
400 bp
D
Vector
ARID2
E
***
120
90
60
30
0
Relative cell migration (%)
Vector
ARID2

Figure S2

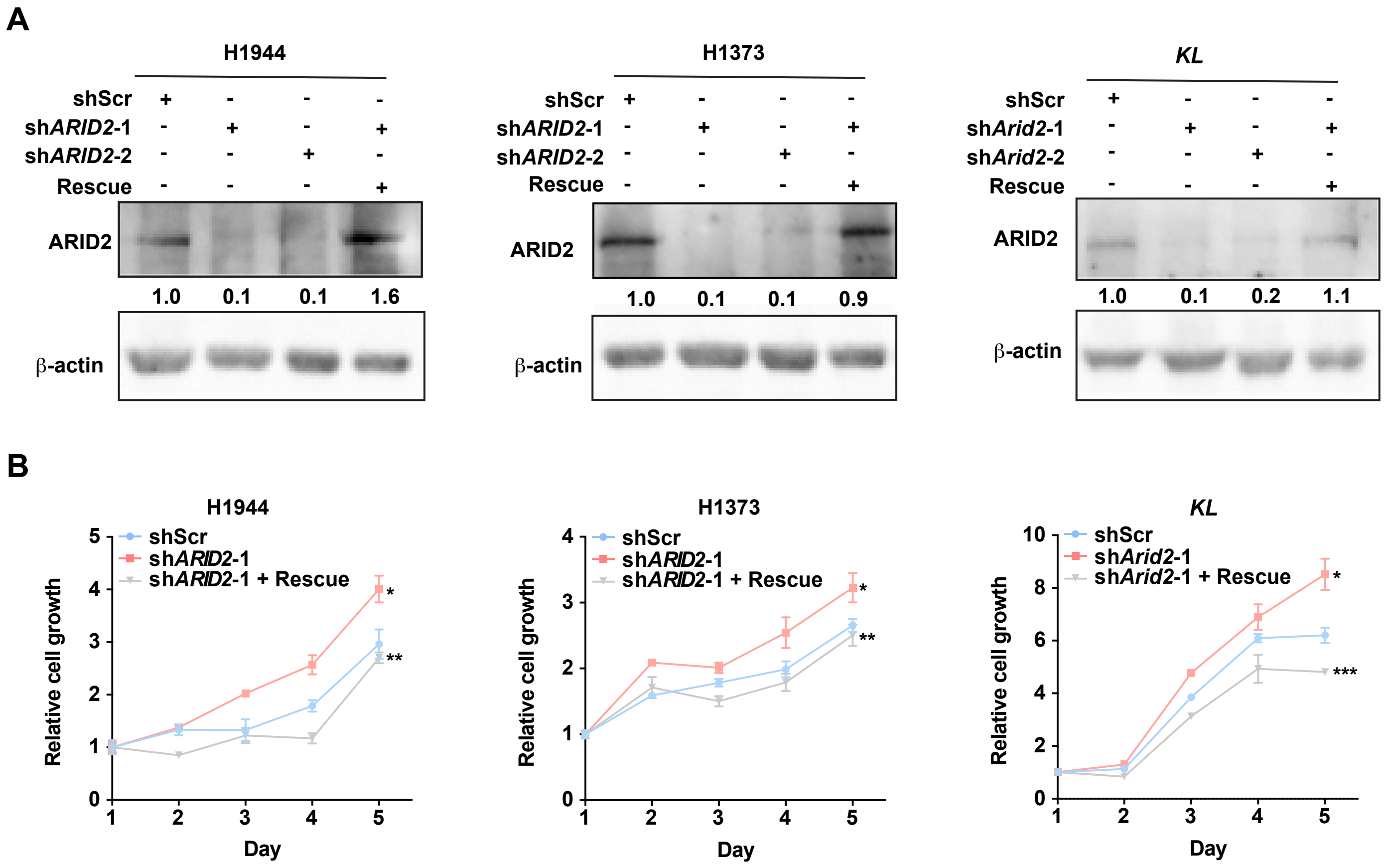
A
H1944
shScr
shARID2-1
shARID2-2
Rescue
ARID2
1.0 0.1 0.1 1.6
β-actin
H1373
1.0 0.1 0.1 0.9
KL
shArid2-1
shArid2-2
1.0 0.1 0.2 1.1
B
Relative cell growth
Day
shARID2-1 + Rescue
shArid2-1 + Rescue

Figure S3

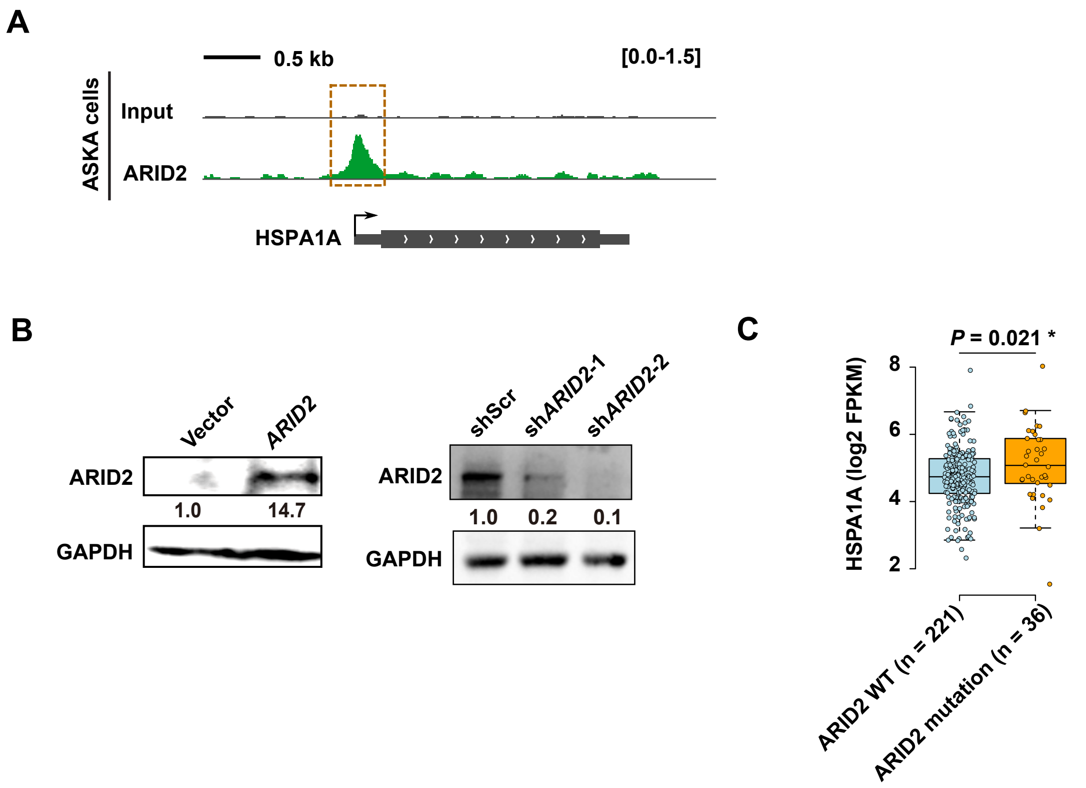
A
0.5 kb
[0.0-1.5]
ASKA cells
Input
ARID2
HSPA1A
B
Vector
ARID2
ARID2
1.0
14.7
GAPDH
shScr
shARID2-1
shARID2-2
ARID2
1.0
0.2
0.1
GAPDH
C
P = 0.021 *
HSPA1A (log2 FPKM)
8
6
4
2
ARID2 WT (n = 221)
ARID2 mutation (n = 36)

Figure S4

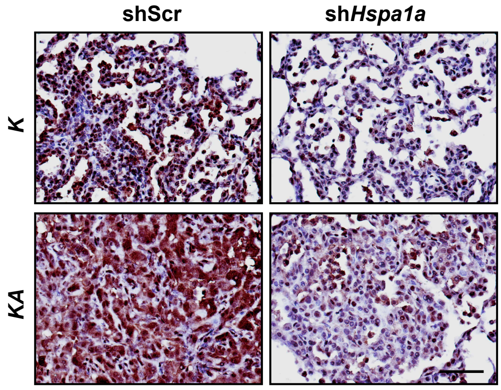

Figure S5

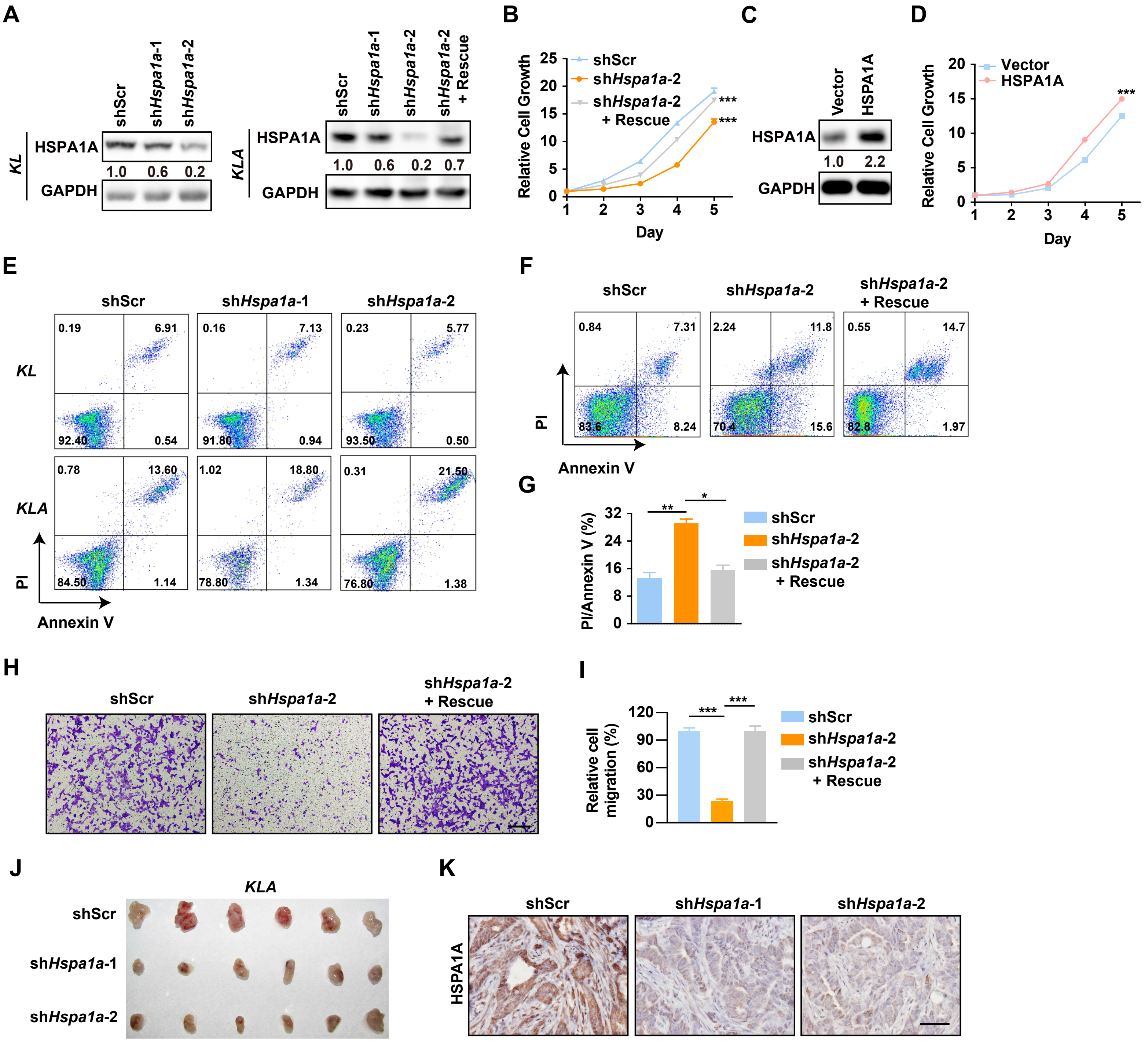
A
shScr
shHspa1a-1
shHspa1a-2
KL
HSPA1A
1.0 0.6 0.2
GAPDH
shScr
shHspa1a-1
shHspa1a-2
shHspa1a-2 + Rescue
KLA
HSPA1A
1.0 0.6 0.2 0.7
GAPDH
B
Relative Cell Growth
Day
shScr
shHspa1a-2
shHspa1a-2 + Rescue
***
***
C
Vector
HSPA1A
HSPA1A
1.0 2.2
GAPDH
D
Relative Cell Growth
Day
Vector
HSPA1A
***
E
shScr
shHspa1a-1
shHspa1a-2
KL
0.19 6.91 92.40 0.54
0.16 7.13 91.80 0.94
0.23 5.77 93.50 0.50
KLA
0.78 13.60 84.50 1.14
1.02 18.80 78.80 1.34
0.31 21.50 76.80 1.38
PI
Annexin V
F
shScr
shHspa1a-2
shHspa1a-2 + Rescue
0.84 7.31 83.6 8.24
2.24 11.8 70.4 15.6
0.55 14.7 82.8 1.97
PI
Annexin V
G
PI/Annexin V (%)
shScr
shHspa1a-2
shHspa1a-2 + Rescue
**
*
H
shScr
shHspa1a-2
shHspa1a-2 + Rescue
I
Relative cell migration (%)
shScr
shHspa1a-2
shHspa1a-2 + Rescue
***
***
J
KLA
shScr
shHspa1a-1
shHspa1a-2
K
shScr
shHspa1a-1
shHspa1a-2
HSPA1A

Figure S6

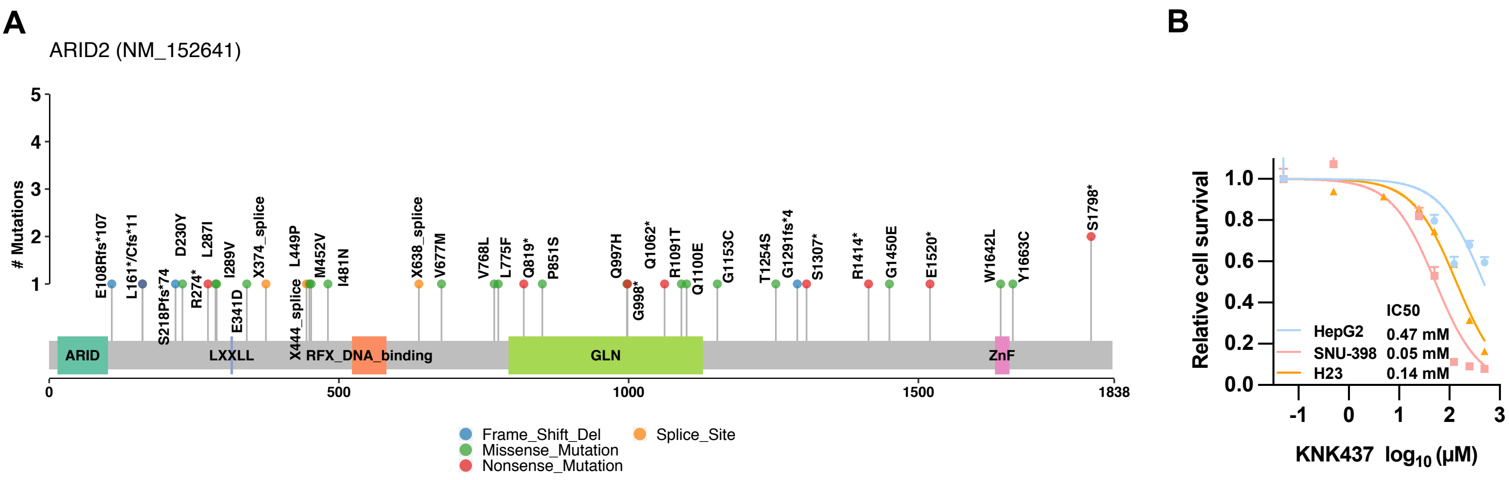
A
ARID2 (NM_152641)
Mutations
5
4
3
2
1
E108Rfs*107
L161*/Cfs*11
S218Pfs*74
D230Y
R274*
L287I
I289V
E341D
X374_splice
X444_splice
L449P
M452V
I481N
X638_splice
V677M
V768L
L775F
Q819*
P851S
Q997H
G998*
Q1062*
R1091T
Q1100E
G1153C
T1254S
G1291fs*4
S1307*
R1414*
G1450E
E1520*
W1642L
Y1663C
S1798*
ARID
LXXLL
RFX_DNA_binding
GLN
ZnF
0
500
1000
1500
1838
Frame_Shift_Del
Missense_Mutation
Nonsense_Mutation
Splice_Site
B
Relative cell survival
1.0
0.8
0.6
0.4
0.2
0.0
-1
0
1
2
3
KNK437 log10 (μM)
IC50
HepG2 0.47 mM
SNU-398 0.05 mM
H23 0.14 mM

Figure S7

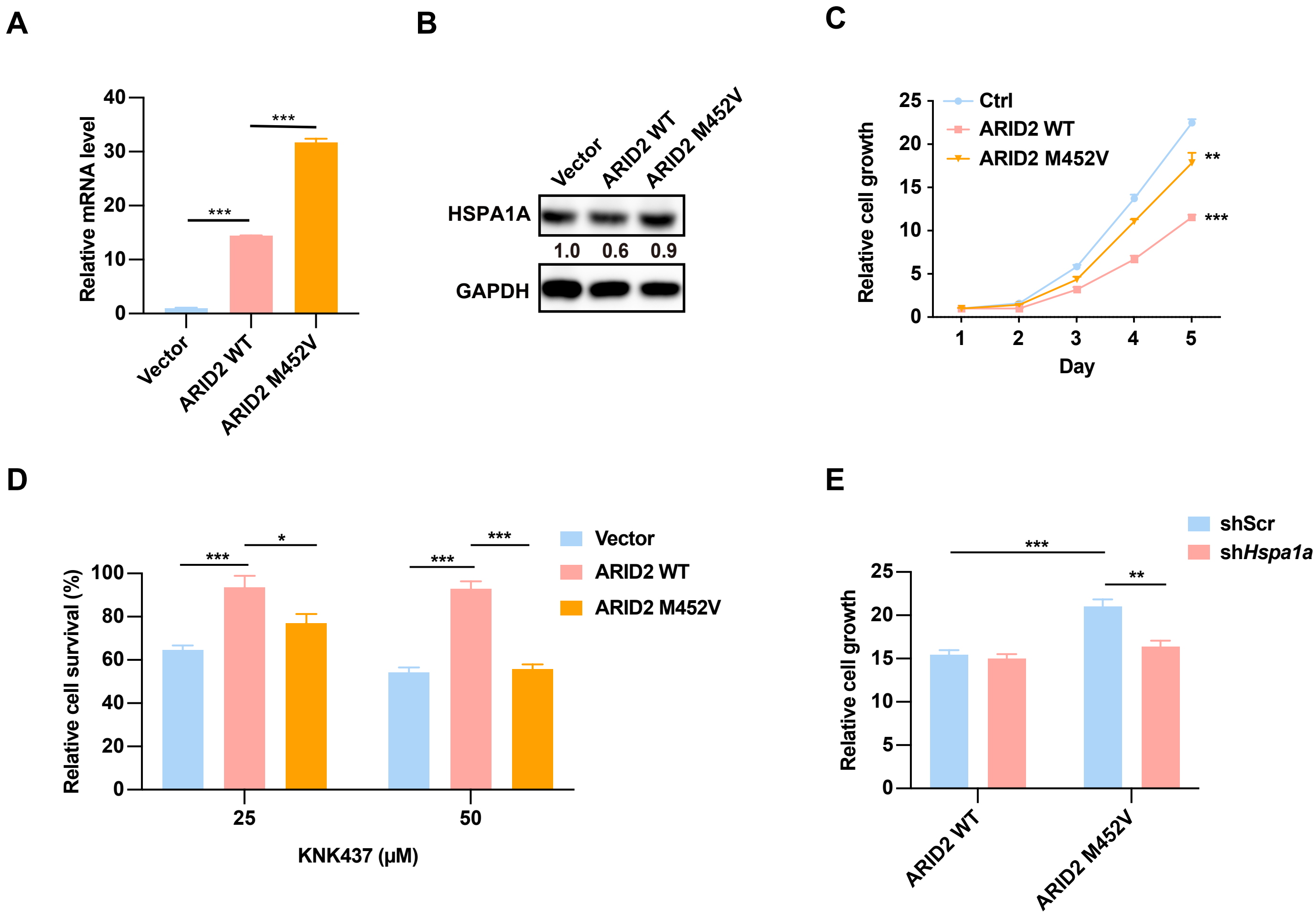
A
Relative mRNA level
40
30
20
10
0
***
***
Vector
ARID2 WT
ARID2 M452V
B
Vector
ARID2 WT
ARID2 M452V
HSPA1A
1.0 0.6 0.9
GAPDH
C
Relative cell growth
25
20
15
10
5
0
Ctrl
ARID2 WT
ARID2 M452V
**
***
1 2 3 4 5
Day
D
Relative cell survival (%)
100
80
60
40
20
0
***
*
***
***
Vector
ARID2 WT
ARID2 M452V
25
50
KNK437 (μM)
E
Relative cell growth
25
20
15
10
5
0
***
**
shScr
shHspa1a
ARID2 WT
ARID2 M452V

Figure S8

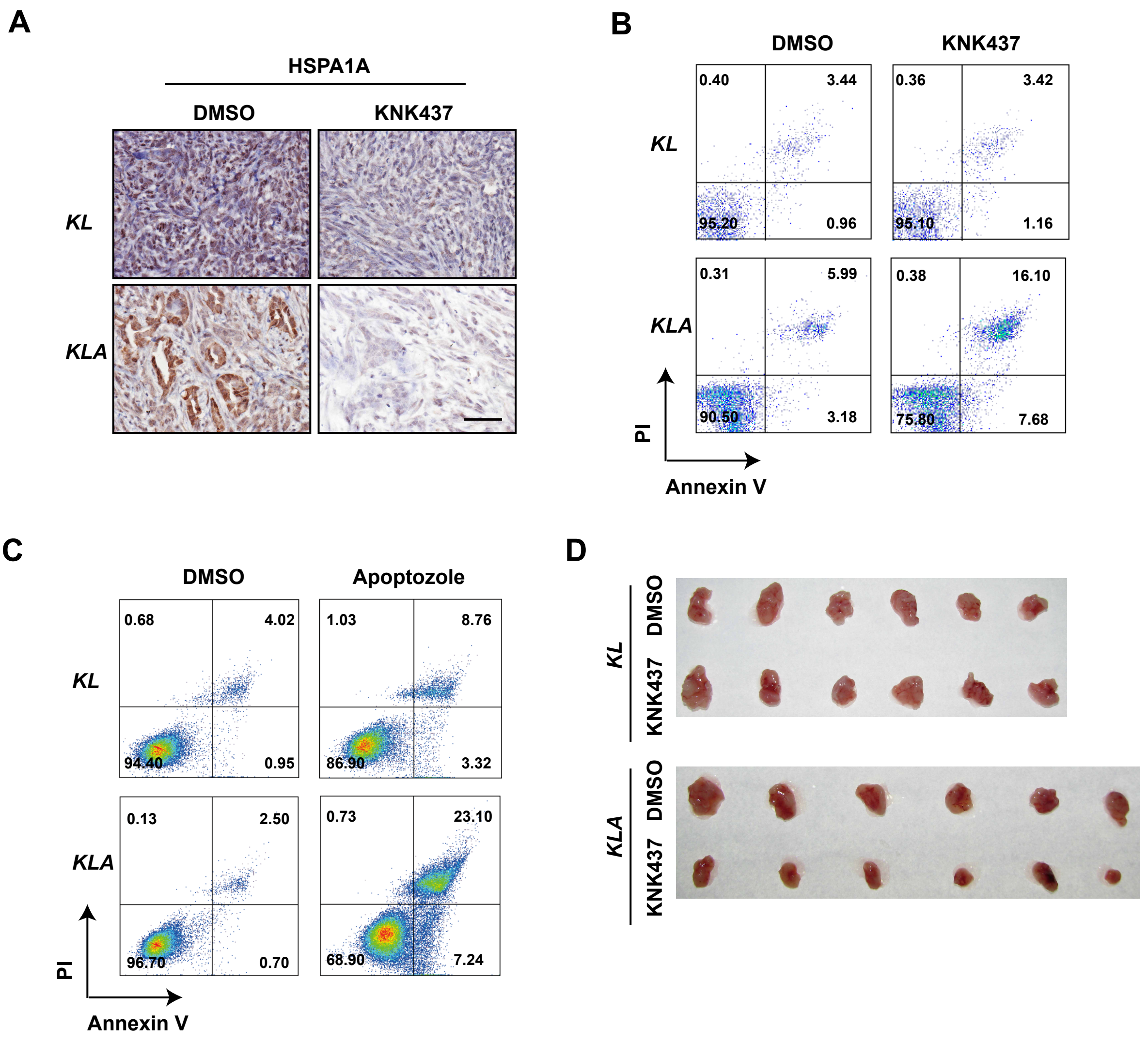
A
HSPA1A
DMSO
KNK437
KL
KLA
B
DMSO
KNK437
KL
0.40
3.44
95.20
0.96
0.36
3.42
95.10
1.16
KLA
0.31
5.99
90.50
3.18
0.38
16.10
75.80
7.68
PI
Annexin V
C
DMSO
Apoptozole
KL
0.68
4.02
94.40
0.95
1.03
8.76
86.90
3.32
KLA
0.13
2.50
96.70
0.70
0.73
23.10
68.90
7.24
PI
Annexin V
D
KL
DMSO
KNK437
KLA
DMSO
KNK437

Figure S9
